# What is category theory to cognitive science? Compositional representation and comparison

**DOI:** 10.3389/fpsyg.2022.1048975

**Published:** 2022-11-18

**Authors:** Steven Phillips

**Affiliations:** Mathematical Neuroscience Group, Human Informatics and Interaction Research Institute, National Institute of Advanced Industrial Science and Technology, Tsukuba, Japan

**Keywords:** category theory, category, functor, natural transformation, analogy, matrix reasoning

## Abstract

Category theorists and cognitive scientists study the structural (analogical) relations between domains of interest albeit in different contexts, that is, formal and psychological systems, respectively. Despite this basic commonality, very few cognitive scientists take a category theory approach toward understanding the structure of cognition which raises the question, What is category theory to cognitive science? An answer is given as the slogan “Category theory is to cognitive science as functor is to representation; as natural transformation is to comparison” to make category theory more accessible and informative for cognitive scientists.

## 1. Introduction

What is category theory to cognitive science? A short answer is that both fields are about “comparison of (compositional) structure” albeit in different contexts. Category theory was invented to formalize correspondences between mathematical constructions (Eilenberg and Mac Lane, 1945; Mac Lane, 1998). Cognitive scientists often view cognition in terms of representations that preserve entity relationships (structure) *via* relationships between corresponding mental states: e.g., *classical compositionality* (Fodor and Pylyshyn, 1988). Despite contextual differences, category theory ideas relate to concepts in cognitive science in ways not generally recognized by cognitive scientists. Some basic connections between a mathematical theory of structure (i.e., category theory) and the structure of cognition are educed here for the purpose of making category theory more accessible and informative to cognitive scientists.

**Figure 1:**
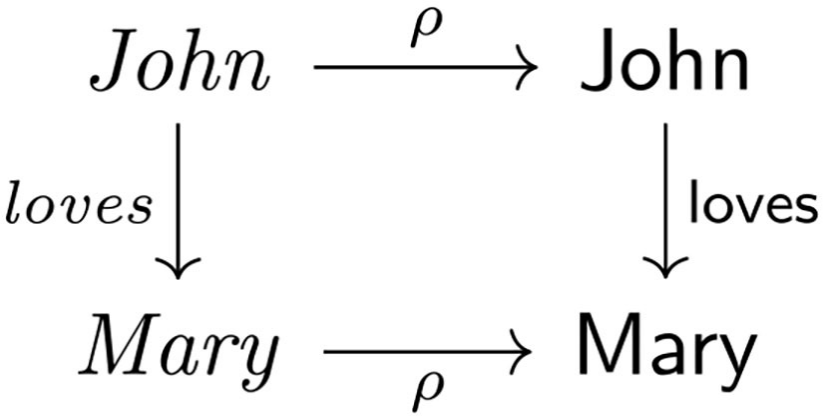
The expression “John loves Mary” as a commutative square of arrows.

One might say that category theory and cognitive science share a common ideal: the representation and comparison of (compositionally) structured entities in some domain of interest. This situation is illustrated as the following square of arrows for the expression, *John loves Mary* (Figure 1). The relationship between the phrase and the concept is depicted as transporting or transforming the left vertical arrow, representing the structure of the expression, to the right vertical arrow, representing the conceptual structure, by sliding along the horizontal arrows, thus forming a square. This arrangement constitutes a so-called *commutative square* in that the chain of arrows in the anticlockwise direction equals the chain of arrows in the clockwise direction, that is, ρ◦*loves* = loves◦ρ, where ◦ signifies the operation for combining arrows to form arrows. This arrangement is reminiscent of the *compositionality principle* (see, e.g., Janssen, 1997; Coecke et al., 2010) in linguistics linking syntax to semantics, or the *structure mapping theory* (Gentner, 1983) of analogy in cognitive psychology as a map from a source domain of knowledge to a target domain of knowledge, which features in a variety of analogy models (Gentner and Forbus, 2011) and a model of metaphor (Fuyama et al., 2020).

Despite the abstract nature of category theory, a substantial amount of the theory organizes around this notion of (typed) commutative square. In the context of cognition, basic category theory constructions correspond to compositional representation and comparison or transformation, which comports with the *representational/computational theory of mind* (Wilson, 1999) whereby cognition is seen as a system of computational processes over cognitive representations (see, Fodor, 1985, for a survey of this and other views). The purpose of this article is to make this consilience more concrete for cognitive scientists. The rest of this introduction is a preview to prime the details that follow.

**Figure 2:**
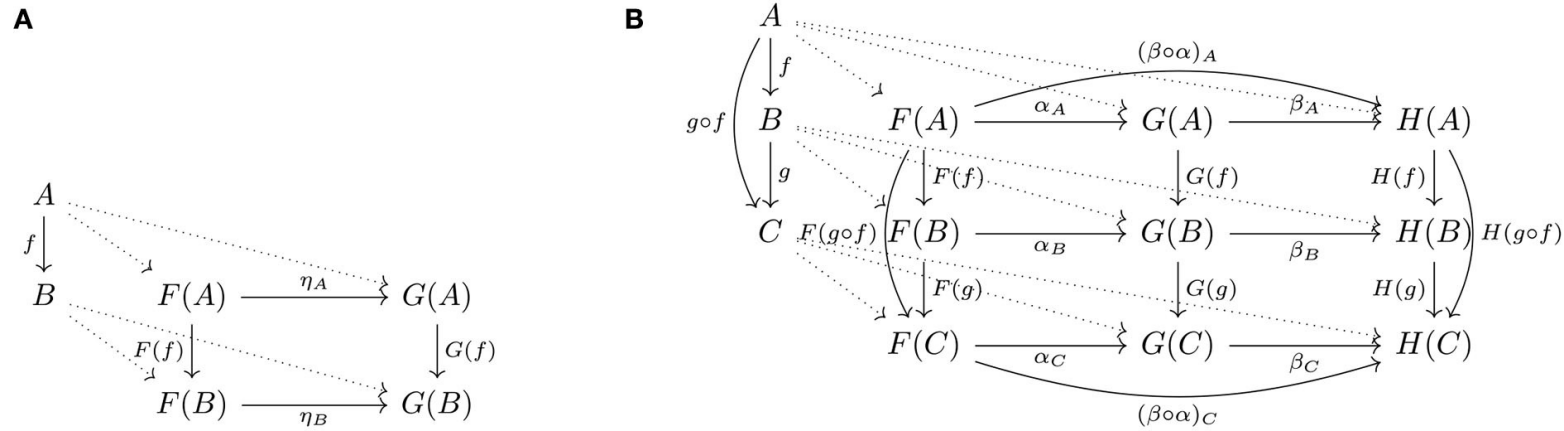
Natural transformations of **(A)** arrows and **(B)** compositions of arrows.

Category theory vastly formalizes this simple idea of preserving structure as commutative squares with applications well beyond mathematics (see, e.g., Fong and Spivak, 2019). In recent years, researchers with a common interest in interdisciplinary applications of category theory have coalesced as the field known as *Applied Category Theory*. This “square” of arrows appears in many guises, historically, beginning with the formal concept of *natural transformation* (Eilenberg and Mac Lane, 1945), which depends on the concepts of *functor* and *category*. The relationships between these concepts are depicted as a diagram of arrows (Figure 2A). A category consists of *objects* and (directed) relations between objects, called *arrows*: e.g., *f* is an arrow from an object *A* to an object *B*, also written *f*:*A*→*B*, in some category **C**. The dotted lines indicate the actions of two functors on the objects and arrows in **C**, that is, respectively, a functor *F* sends *A*, *B*, and *f* to objects *F*(*A*) and *F*(*B*) and arrow *F*(*f*) in some category **D** and a functor *G* sends *A*, *B*, and *f* to the objects *G*(*A*) and *G*(*B*) and arrow *G*(*f*) also in **D**. Functors are arrows between objects that are categories: e.g., *F, G*:**C**→**D**. The objects *F*(*A*) and *F*(*B*) and the arrow *F*(*f*) constitute the *image* of *F*; likewise, the objects *G*(*A*) and *G*(*B*) and the arrow *G*(*f*) constitute the image of *G*. The (naturality) square of arrows involving the images of *F* and *G* depicts a *natural transformation*, that is, a map η from *F* to *G*, written $\eta :F\overset{.}{\to }G$, consisting of a component map η_*A*_:*F*(*A*) → *F*(*A*) for each object *A* in **C** such that for each arrow *f* in **C** the square commutes. Thus, natural transformations are maps between functors and functors are maps between categories, hence the logical dependencies. Although originally introduced for applications in topology (Eilenberg and Mac Lane, 1945), category theory constructions also feature in diverse fields such as the use of natural transformations in music (Mannone and Favali, 2019) and categorical forms of compositionality in aesthetics (Kubota et al., 2017).

Categories, functors, and natural transformations partake in another important aspect of the general theory—*compositionality*: the composition of two *compatible* arrows (i.e., a pair of arrows linked “head to tail”) is an arrow, for example, *f*:*A*→*B* composed with *g*:*B*→*C* is *g*◦*f*:*A*→*C*, where again ◦ signifies the composition operation. Composition operates at all levels: arrows between objects, functors between categories and natural transformations between functors. The diagram of arrows (Figure 2B) involves composition of arrows, *f*:*A*→*B* and *g*:*B*→*C*, the action of functors on composed arrows, *F*(*g*◦*f*), and composition of natural transformations, $\alpha :F\overset{.}{\to }G$ and $\beta :G\overset{.}{\to }H$. (The diagram does not show functor composition, that is, in the third, out-of-plane direction.) These apparently different forms of compositionality are actually the same concept in different contexts (or dimensions): ordinary arrows are arrows between ordinary objects (vertical dimension), functors are arrows between objects that are categories, and natural transformations are arrows between objects that are functors in a category of functors (horizontal dimension). Natural transformations also compose with functors. Category theory provides a vast generalization of the notion of compositionality that is relevant to cognitive science.

**Figure 3:**
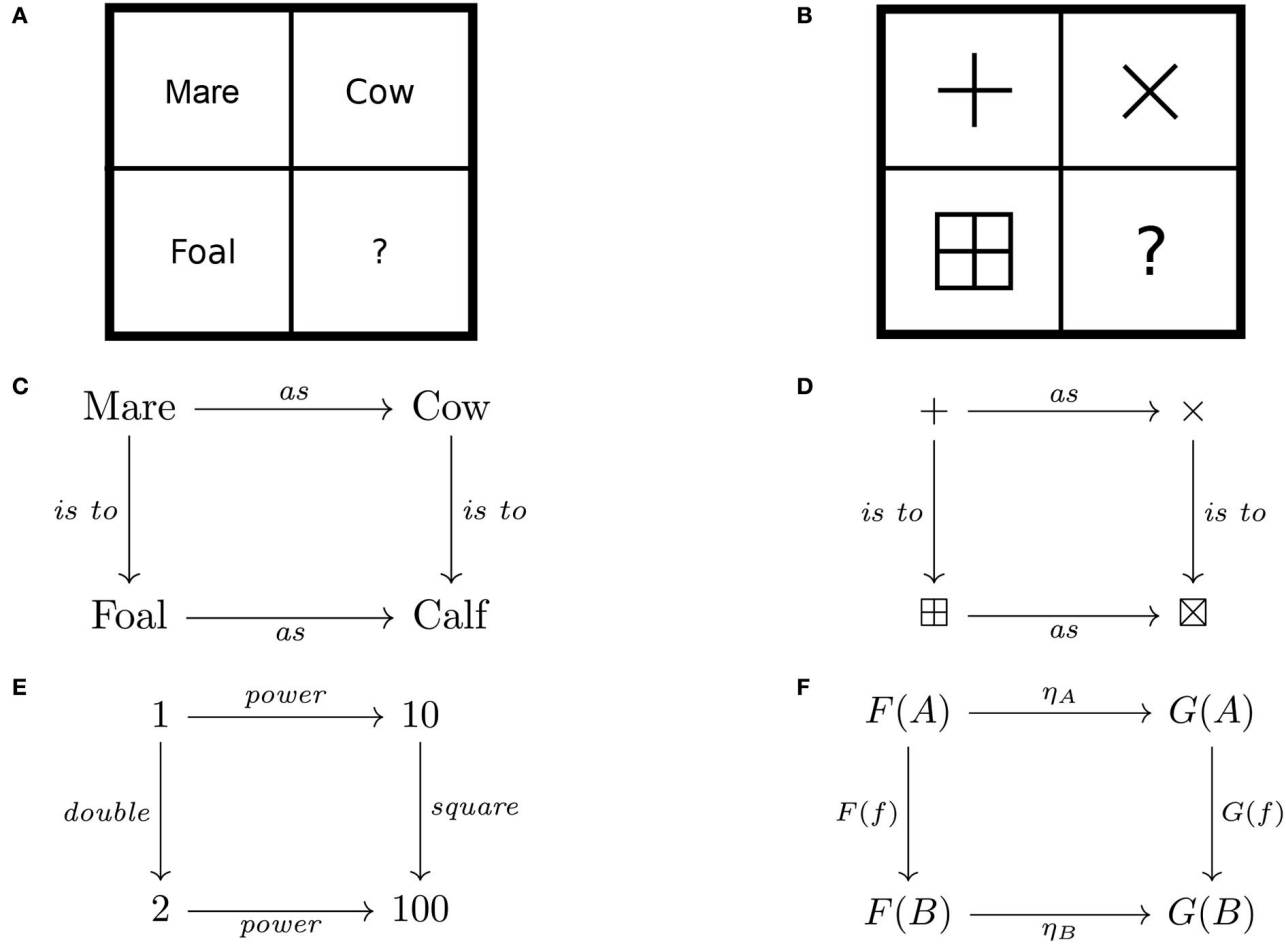
A comparison of proportional analogy, matrix reasoning, and natural transformation: **(A)** “Mare is to foal as cow is to calf” and **(B)** “+ is to ⊞ as × is to ⊠” in matrix form, and **(C,D)** in the form of a natural transformation. Algebraic relations, for example, **(E)** the power function are also instances of natural transformations, whose general form is given as **(F)** a commutative square of arrows.

Basic category theory concepts, though straightforwardly introduced this way, engender little intuition regarding applications as nothing is said about their specific nature. For cognitive scientists, however, these squares of relations between objects are also reminiscent of the squares of stimuli used in matrix reasoning tasks (Raven et al., 1998), which have been studied as tests of intelligence (Carpenter et al., 1990). Such reasoning tasks involve a matrix of stimuli with a missing cell that can be completed by applying the relationship educed from the rows or columns with all cells filled. For instance, the proportional analogy *Mare is to foal as cow is to what?* in matrix form involves a two-by-two matrix. The empty cell is filled with *calf* by educing that the relevant relationship between *mare* and *foal* is *gives-birth-to* and applying this relationship to *cow* to obtain *calf* . These and other such matrices of stimuli have a common form symbolized by the expression *a*:*b*::*c*:*d*, where the semicolon corresponds to relationships in one (say, vertical) direction and the double semicolon to relationships in the other (horizontal) direction. This situation is analogous to a commutative square (Figure 3), which naturally extends to matrices with more rows and columns (cf. Figure 2B). Such comparisons afford a perspicuous way of bootstrapping intuitions about category theory concepts (Section 2) for potential applications in cognitive science (Section 3) to complement other conceptualizations (see, e.g., Phillips, 2021a, and the references therein).

Some readers may ponder the need for detailed explanations of basic category theory concepts given the many introductions that already exist. However, category theory introductions typically presume a style of thinking that can bedevil those outside the target audience seeking intuition (see Lawvere and Schanuel, 2009, for a general readership). For those readers, the theory can appear as a bridge to nowhere. Yet mathematics is arguably as much a reflection of thinking as it is about the world (Mac Lane, 1986; Lakoff and Núñez, 2000). The purpose here is to present category theory concepts to cognitive scientists in a way that enables thinking about thinking, categorically. Accordingly, this presentation departs from the usual style of relegating technical details to an appendix in favor of a side-by-side comparison to facilitate understanding—the devil is in the *comparable* details—which affords a novel synthesis of concepts for the purpose of doing cognitive science.

## 2. Some formal and conceptual comparisons

The formal basis of comparison employed here is the concept of natural transformation, which depends on the concept of functor, which in turn depends of the concept of category. Accordingly, categories are introduced first (Section 2.1), followed by functors (Section 2.2), and then natural transformations (Section 2.3).

### 2.1. Categories and compositional structure

Both category theory and cognitive science are concerned with modeling the *(compositional) structure* of some “domain” of interest, that is, the entities, the entity relationships, and the way that combinations for those relationships are themselves related. (The term domain is used in two senses: informally, as a topic of interest and, formally, as the source object of an arrow, Section 2.1.2). In cognitive science, for example, one may regard a cognitive process as consisting of a chain of subprocesses, a cognitive system as composed of subsystems, or a cognitive representation of some complex entity as constructed from representations of constituent entities. Category theory also considers a wide variety of compositional forms, including composition of functions, relations, algebraic structures, and spaces. All forms are based on a concept of compositionality, introduced here, that also pertains to situations of interest to cognitive scientists.

The concept of a category depends on several constituent concepts. Briefly, a category consists of a collection of *objects* and *arrows* (Section 2.1.1) where each arrow is directed from its *domain* object to its *codomain* object (Section 2.1.2), including self-directed *identity arrows* (Section 2.1.3), that combine as arrows by a *composition operation* satisfying certain properties (Section 2.1.4). A formal definition of category (Section 2.1.5) captures the notion of structure as an arrow: The collection of such structures is a category of such arrows (now objects), and the arrows between these objects are structure-preserving maps given as commutative squares (Section 2.1.6).

#### 2.1.1. Elements are related as objects and arrows

A domain of interest (such as a group of people) is typically considered in terms of its elements and relationships between those elements. An ordered set is a simple example of how one might model a domain this way. Suppose, for instance, three people of interest, *Ann, Bob*, and *Cal*, and that *Ann is shorter than Bob* and *Bob is shorter than Cal*. This domain can be modeled as an *ordered set*, that is, the set of people *P* = {*Ann, Bob, Cal*} together with their order relationships *Ann* < *Bob* and *Bob* < *Cal*. These elements and their order relationships correspond to instances of two basic kinds of constituents for a category, that is, each element corresponds to an instance of an abstract notion of entity, called *object*, and each order relationship to an instance of an abstract notion of relation between entities (objects), called *arrow* or *morphism* or *map*. So, for instance, the element *Ann* is now an object and the relationship *Ann* < *Bob* is now an arrow *Ann*→*Bob* in some category corresponding to an ordered set.

*Element is to relationship as object is to arrow*.

The simplicity of this example belies several important subtleties with the move from sets to categories that are elaborated here and in subsequent sections. First, notice that the *less-than* symbol “points” to the shorter person whereas the arrow points to the taller person. Relationships are directed. Direction is captured syntactically (diagrammatically) by the direction of each arrow and semantically by two additional relations (maps) between objects and arrows, introduced in the next section (Section 2.1.2). What matters is that the relationship between syntax and semantics is consistent. Compare, for instance, the correspondence between order relationship *Ann*>*Bob* and arrow *Ann*←*Bob*.

Second, in general, order relationships are expressed using the “less-than *or* equals” symbol, ≤ , rather than the less-than symbol. So, for instance, *Ann is shorter than or the same height as Bob*, now written *Ann* ≤ *Bob*, means that *Ann is not taller than Bob*. The significance of this interpretation is that *Ann is not taller than Ann*, that is, the *self-directed* relationship *Ann* ≤ *Ann* which corresponds to the arrow *Ann*→*Ann*. Order relations that are strictly less-than, that is, *a* < *b* but not *a* ≤ *b* (whence, *a* ≰ *a*), are called *strict orders*. For example, *parent-of* is a strict order. The relevance of this distinction to category theory will be elaborated shortly (Section 2.1.3).

Third, there may be more than one relationship between entities, in general, hence more than one arrow between a pair of objects. Notation referencing the arrow and the pair of objects serves to distinguish arrows in a category. Suppose, for instance, *Ann is younger than or the same age as Bob* is expressed by the order relationship *Ann* ⊑ *Bob*, that is, *Ann is not older than Bob*. The arrows corresponding to the two order relationships between *Ann* and *Bob* are referenced in full as ≤_*AB*_:*Ann*→*Bob* and ⊑_*AB*_:*Ann*→*Bob*, that is, different symbols or identifiers are used to distinguish different arrows between the same pair of object.

Fourth, and finally, category theory often affords more than one way to model a domain of interest. For instance, a (binary) relation *R* between sets *A* and *B* can be modeled as a collection of arrows between objects corresponding to the elements partaking in that relationship, as in the *Ann-Bill-Cal* example: there is an arrow *a*→*b* for each element *a* ∈ *A* that is in an *R* relationship with an element *b* ∈ *B*. For this reason, such arrows are referred to as relationships, rather than relations. Or, as we will see later (Section 2.1.5, example 2), *R* can be modeled as a single arrow between those sets, *A*→*B*. The term relation is used when a distinction between relation and relationship is not essential.

#### 2.1.2. Relations are directed as domains before codomains

As mentioned earlier, the direction of each relationship is depicted by the direction of each arrow: e.g., ≤_*AB*_:*Ann*→*Bob* is directed from object *Ann* to object *Bob*, which says that *Ann* comes before *Bob* and *Bob* comes after *Ann*. The objects *Ann* and *Bob* are called the *domain* and the *codomain* of the arrow ≤_*AB*_, respectively.

*Before is to after as domain is to codomain*.

Expressions ≤_*AB*_:*Ann*→*Bob* and ≤_*AB*_:*Bob*←*Ann* identify the same arrow, whose directionality is determined by two maps between objects and arrows. In general, for a category **C**, the collection of **C**-objects is denoted **C**_0_ and the collection of **C**-arrows is denoted **C**_1_. Two maps from arrows to objects *dom*:**C**_1_→**C**_0_ and *cod*:**C**_1_→**C**_0_ determine the domain and codomain object of each arrow, respectively. In the *Ann-Bob-Cal* example, the corresponding category, denoted *ABC*, consists of the set of objects *ABC*_0_ = {*Ann, Bob, Cal*}, the set of arrows *ABC*_1_ = { ≤_*AB*_, ≤_*BC*_, … } and mappings that include *dom*: ≤_*AB*_↦*Ann* and *cod*: ≤_*AB*_↦*Bob*.

Notice that nothing is said about the nature of object and arrow beyond their relationship to each other. How one is supposed to interpret these formal concepts depends on context, that is, the category in which they reside. For instance, in the context of sets and functions, an object is a set and an arrow is a function. Category theory can been seen as an abstraction of set theoretical constructions; hence, the notation and nomenclature are often taken from there: e.g., arrows (morphisms, maps) are generally written *f*:*A*→*B* even though objects need not be sets and arrows need not be functions (homomorphisms, mapping elements in a way that preserves their relationships).

#### 2.1.3. Self-directed relations as identity arrows

As also mentioned earlier, order relationships are typically expressed using the ≤ symbol. Thus, *A* ≤ *B* says that *A* comes no later than *B*. For the *Ann-Bob-Cal* example, this situation means that each person is ordered with respect to themselves: e.g., *Ann* ≤ *Ann* means that *Ann* is not taller than herself. Hence, the set of arrows for the corresponding category, *ABC*, includes the self-directed arrows *Ann*→*Ann*, *Bob*→*Bob* and *Cal*→*Cal*. A relation *R* on *A* is called *reflexive* (has the reflexivity property) if every element *a* ∈ *A* is related to itself. Reflexivity of order corresponds to an arrow ≤_*A*_:*A*→*A* for each object *A* in some ordered set as a category. The arrow ≤_*A*_:*A*→*A* is called the *identity arrow* at *A*.

*Relationship is to reflexivity as arrow is to identity*.

Every object *A* in a category is associated with an identity arrow, written 1_*A*_:*A*→*A*. For example, in the context of sets and functions, the identity arrow at set *A* is the identity function 1_*A*_:*a*↦*a*. The reason for denoting the identity arrow as 1_*A*_ is by analogy to multiplication of a number by 1 (Section 2.1.4). A self-directed arrow need not be an identity arrow. For instance, the constant function on a set *A* sending every element *a* ∈ *A* to the same element *k* ∈ *A*, that is, *f*:*A*→*A*; *a*↦*k*, is self-directed but not an identity function. The composition of two compatible arrows is an arrow (Section 2.1.4). The rules for composition imply that every object in a category is associated with one and only one identity arrow. Thus, for a category **C**, there is another relation between objects and arrows that is given by the map *id*:**C**_0_→**C**_1_; *A*↦1_*A*_.

The implication that every object is associated with one identity arrow may seem too restrictive for cognitive science in situations where the entities do not have self-directed relationships and thus lack a meaningful interpretation in terms of the identity arrows of a category. However, as explained later (Section 2.1.6), these situations can be modeled by other categories.

#### 2.1.4. Transitivity of relations as composition of arrows

Order relationships are themselves related to each other in a way that is called *transitivity*. For instance, *Ann is shorter than Bob* and *Bob is shorter than Cal* implies *Ann is shorter than Cal*. All triples of ordered elements are related this way, that is, the transitivity property of order relations. Formally, a relation *R* between two sets *A* and *B* is a subset of the set of all pairs of elements with the first element of each pair drawn from *A* and the second element of each pair drawn from *B*, called the *Cartesian product* of *A* and *B*, that is, the set *R*⊆*A*×*B* = {(*a, b*)|*a* ∈ *A, b* ∈ *B*}. If the pair (*a, b*) is in *R*, then we say that *a* is *R-related* to *b*, or write *aRb* to indicated this relationship. A relation *R* on *A*, that is, a subset of *A*×*A*, is called *transitive* (has the transitivity property) if *aRa*′ and *a*′*Ra*″ implies *aRa*″ for all triples of elements *a, a*′, *a*″ in *A*. Transitivity of order corresponds to conjunction of arrows: If there is an arrow *A*→*B* *and* an arrow *B*→*C*, then there is a *composite* arrow *A*→*C*. This conjunction of arrows is a form of *composition*.

*Relationship (order) is to transitivity as arrow is to composition (conjunction)*.

In any category **C**, if *f*:*A*→*B* and *g*:*B*→*C* are a pair of *compatible* arrows, that is, the codomain of the first arrow, *f*, is the domain of the second arrow, *g*, then there is a composite arrow from *A* to *C* in **C**, written *g* ◦ *f*:*A*→*C*, where ◦ denotes the *composition operation*, simply called *composition*. Composition is a (partially defined) map sending each pair of compatible arrows to their composition, that is, the map *comp*:**C**_1_×**C**_1_→**C**_1_; (*f, g*)↦*g* ◦ *f*, also denoted ◦ (−, −), cf. expressions +(1, 2) and 1+2. For sets and functions, composition of compatible functions *f*:*A*→*B* and *g*:*B*→*C* is the composite function *g* ◦ *f*:*A*→*C*; *a*↦*g*(*f*(*a*)) mapping each element *a* ∈ *A* to the element *g*(*f*(*a*)) ∈ *C*, read *g of f of a*, hence the notational order. Note that in this context (category) of sets and functions, composition is a function *on* functions sending *f* and *g* to some function *h* in that category, that is, *g* ◦ *f* = *h*, cf. 1+2 = 3. The corresponding form for composition of order arrows ≤_*AB*_:*A*→*B* and ≤_*BC*_:*B*→*C* is ≤_*AC*_ = ≤_*BC*_ ◦ ≤_*AB*_:*A*→*C*, where composition takes on the role of conjunction. In this context (ordered set as a category), composition is a function *on* order relationships. This situation compares with *transitive inference* as the logical rule of replacement *aRb* ∧ *bRc*⇒*aRc*, where ∧ is conjunction.

Composition of orders and functions satisfy two important properties that are required of a composition operation on the collection of arrows in any category, generally: *associativity* (Section 2.1.4) and *unity* (section 2.1.4).

##### 2.1.4.1. Composition is associative: Composition order is commutative

Suppose the *Ann-Bob-Cal* example is extended to include a fourth person, *Dan*, and the order relationship *Cal is shorter than Dan*. Transitive inference can be applied twice in two ways to infer than *Ann is shorter than Dan*: (1a) *Ann* ≤ *Bob* and *Bob* ≤ *Cal* implies *Ann* ≤ *Cal* and (1b) *Ann* ≤ *Cal* and *Cal* ≤ *Dan* implies *Ann* ≤ *Dan*, or (2a) *Bob* ≤ *Cal* and *Cal* ≤ *Dan* implies *Bob* ≤ *Dan* and (2b) *Ann* ≤ *Bob* and *Bob* ≤ *Dan* implies *Ann* ≤ *Dan*. Compare this logical equivalence with the equality of the corresponding arrows, that is, a comparison of

- *aRb* ∧ (*bRc* ∧ *cRd*)⇔(*aRb* ∧ *bRc*) ∧ *cRd* and
- ≤_*CD*_ ◦ (≤_*BC*_ ◦ ≤_*AB*_) = (≤_*CD*_ ◦ ≤_*BC*_) ◦ ≤_*AB*_.

The order of compositions does not affect the result, that is, conjunction is *associative*. Associativity is also a property of addition, that is, *x*+(*y*+*z*) = (*x*+*y*)+*z*.

*Associativity is to addition as associativity is to composition*.

In any category **C**, the composition operation is associative, that is, *h* ◦ (*g* ◦ *f*) = (*h* ◦ *g*) ◦ *f* for all triples of compatible arrows *f, g, h* in **C**. Brackets can be omitted, since the result is not affected by order of composition—cf. *h* ◦ *g* ◦ *f* and *x*+*y*+*z*.

**Figure 4:**
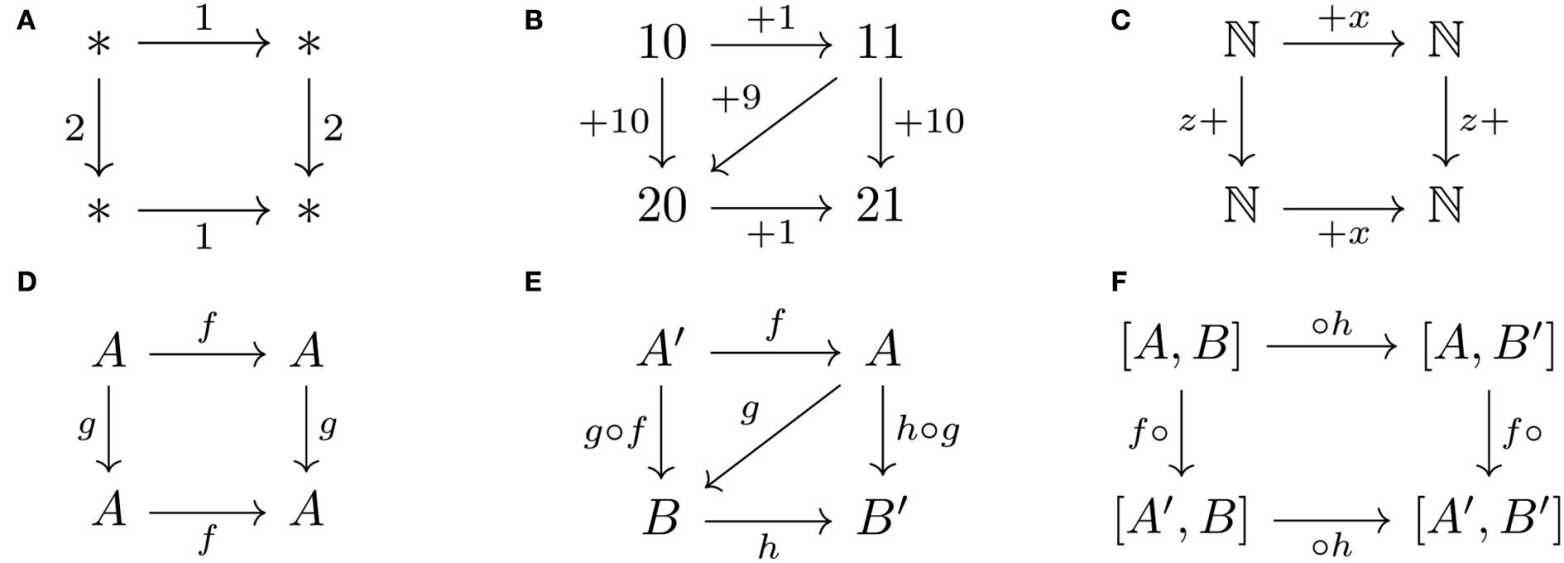
Commutativity and associativity properties are expressed as commutative squares: **(A)** commutativity of addition, **(B,C)** associativity of addition, **(D)** commutative composition operation, and **(E,F)** associativity of composition operation.

Associativity of composition is essentially commutativity of composition order (Figure 4). The “commutativity” of commutative squares is analogous to the commutativity of addition given as a square of arrows corresponding to numbers (Figure 4A). Addition can also be expressed as a square of operations between numbers (Figure 4B) or set of numbers (Figure 4C). A commutative composition operation is analogous to the commutativity of addition (Figure 4D), but composition is generally not commutative. However, associativity of composition can be expressed as a commutative square (Figure 4E), which in turn is expressed as a commutative square of operations on (hom-)sets of arrows (Figure 4F). Thus, the order of composition is commutative, hence the analogy between associativity and commutativity.

*Commutativity is to addition as associativity is to order of composition*.

##### 2.1.4.2. Composition with identity arrows is unital

Identity arrows, introduced earlier (Section 2.1.3), play a special role with respect to composition just like the number 1 plays a special role with respect to multiplication.

*One is to multiplication as identity is to composition*.

Self-directed order arrows, ≤_*A*_, also play an analogous role. Compare

- *x*×1 = *x* = 1 × *x*,
- ≤_*AB*_ ◦ ≤_*A*_ = ≤_*AB*_ = ≤_*B*_ ◦ ≤_*AB*_ and
- *f* ◦ 1_*A*_ = *f* = 1_*B*_ ◦ *f*.

For any category **C**, the composition operation has this property, called *unity*, that is, *f* ◦ 1_*A*_ = *f* = 1_*B*_ ◦ *f* for all arrows *f* in **C**.

#### 2.1.5. Formal constructions formalized as categories

The previous sections introduced all the basic concepts that make up a formal definition of category (Definition 1), that is, the concepts of object, arrow, domain, codomain, identity arrow, composition operation and the associativity, and unity properties for composition. A formal definition of category is introduced next followed by examples that further exercise this concept.

** Definition 1 (category)**. *A *category* **C** = (**C**_0_, **C**_1_, *dom, cod, id*, ◦ ) consists of*

- a collection of *objects*, **C**_0_ = {*A, B, C*, … },
- a collection of *arrows*, **C**_1_ = {*f, g, h*, … }—an arrow *f* is directed from an object *A* to an object *B*, written *f*:*A*→*B*, called the *domain* and *codomain* of *f*, respectively,
- two maps *dom, cod*:**C**_1_→**C**_0_ sending each arrow *f*:*A*→*B* to its domain and codomain object, respectively, that is, *dom*(*f*) = *A* and *cod*(*f*) = *B*,
- a map *id*:**C**_0_→**C**_1_ assigning to each object *A* an arrow 1_*A*_:*A*→*A*, called the *identity arrow* at *A*, that is, *id*(*A*) = 1_*A*_, and
- a *composition operation*, ◦ , sending each pair of *compatible* arrows *f*:*A*→*B* and *g*:*B*→*C*, that is, *cod*(*f*) = *dom*(*g*), to the arrow *g* ◦ *f*:*A*→*C* that is
  – *associative*: *h* ◦ (*g* ◦ *f*) = (*h* ◦ *g*) ◦ *f*, and
  – *unital*: *f* ◦ 1_*A*_ = *f* = 1_*B*_ ◦ *f*

for all compatible arrows *f, g, h* ∈ **C**_1_. The collection of arrows in **C** with domain *A* and codomain *B* is called a *hom-set*, written Hom_**C**_(*A, B*), **C**(*A, B*), or [*A, B*] when the category is clear from context.

Several examples of categories were already given in the previous section to illustrate the basic concepts. These and closely related examples are listed for comparison (Example 2).

**Example 2 (sets, functions, relations)**. *The following are categories*.

1. *The category **Set** has sets for objects and (total) functions between sets for arrows. The identity arrows are the identity functions. Composition is composition of functions*.
2. *Restricting the collection of functions to be inclusions, *A*⊆*B*, yields another category, denoted **Set**^⊆^. The identity arrows are *A*⊆*A**.
3. Every set *S* corresponds to a category whose objects are the elements *s* ∈ *S* and only arrows are the identity arrows 1_*s*_:*s*→*s*. Categories with only identity arrows are called *discrete categories*. Composition is trivial: 1_*s*_ ◦ 1_*s*_ = 1_*s*_. Certain sets play an important role, such as the empty set, ∅, singleton set, written {*} when the identity of the only element is not needed, and the set of natural numbers, ℕ. These sets correspond to important categories.
  *(a) The empty category, denoted **0** (or 0), has no objects or arrows. Composition is the empty map, that is, ∅ × ∅ → ∅. Likewise, *dom*, *cod* and *id* are empty maps*.
  *(b) The singleton category, denoted **1** (or 1), has one object and one (identity) arrow*.
  *(c) An index category, denoted *I*⊆ℕ, has a subset of the natural numbers as objects*.
4. *An ordered set (*P*, ≤) corresponds to a category whose objects are the elements *p* ∈ *P* with an arrow *p*→*q* whenever *p* ≤ *q*. Identity arrows correspond to reflexivity and composition to transitivity*.
5. *The category **Rel** has sets for objects and relations between sets for arrows. The identity arrows are the identity relations, that is, 1_*A*_ = {(*a, a*)|*a* ∈ *A*}. The composition of arrows is defined by the *join* of the corresponding relations, that is, the join of relations *R*⊆*A*×*B* and *S*⊆*B*×*C* is the relation $R⋉S=\{(\overset{`}{\pi }(r),\overset{´}{\pi }(s))|r\in R,s\in S,\overset{`}{\pi }(r)=\overset{´}{\pi }(s)\}$, where $\overset{´}{\pi }\left(a,b\right)=a$ and $\overset{`}{\pi }\left(b^{\prime },c\right)=c$, that is, $\overset{´}{\pi }$ and $\overset{`}{\pi }$ return the left (first) and right (second) elements of each pair, respectively. In other words, a pair (*a, c*) is in the join of *R* and *S* whenever there exists a pair (*a, b*) in *R* and a pair (*b*′, *c*) in *S* such that *b* = *b*′*.

Functions and relations are closely connected. Every function corresponds to a relation, and every relation corresponds to a set-valued function (Remark 3). Relations can be used to model non-determinism: Each element is sent to a set of possible outcomes.

** Remark 3**. The *graph of a function* *f*:*A*→*B* *is the relation* Γ(*f*) = {(*a, f*(*a*))|*a* ∈ *A*)}⊆*A*×*B*. A relation *R*⊆*A*×*B* *corresponds to the set-valued (partial) function *f*_*R*_:*a*↦{*b*|(*a, b*) ∈ *R*} defined on the subset of *R*-related elements of* *A*.

The join of two relations is analogous to compatibility of arrows. Accordingly, composition can be defined as a total function on the pairs of compatible arrows (Remark 4).

** Remark 4**. *For a category **C**, the collection of pairs of compatible arrows is given by a *constrained product* on the collection of arrows, that is, **C**_1_×_**C**_0__**C**_1_ = {(*f, g*)|*cod*(*f*) = *dom*(*g*), *f* ∈ **C**_1_, *g* ∈ **C**_1_}. In this case, composition is the (total) map *comp*:**C**_1_×_**C**_0__**C**_1_→**C**_1_*.

Another alternative defines composition as a family of (total) maps indexed by triples of objects in the category (remark 5).

** Remark 5**. *For a category **C**, the composition of arrows can be defined for each triple of objects *A, B, C* in **C** as the total function* ◦ _*ABC*_:[*A, B*] × [*B, C*] → [*A, C*];(*f, g*)↦*g* ◦ *f*.

Notice that if the triple of objects is (*A, A, A*), then composition is just composition of self-directed arrows, that is, ${◦}_{AAA}:\left[A,A\right]\times \left[A,A\right]\to \left[A,A\right];\left(a,a^{\prime }\right)\mapsto a^{\prime }◦a$. And since composition is associative and unital, we also have *a* ◦ (*a*′ ◦ *a*″) = (*a* ◦ *a*′) ◦ *a*″ and *a* ◦ 1 = *a* = 1 ◦ *a* where 1 is the identity arrow at *A*. As we have already seen, this situation is analogous to multiplication for numbers: e.g., × _ℕ_:ℕ × ℕ → ℕ; (*x, y*)↦*x*·*y*. Multiplication is also associative and unital: *x*·(*y*·*z*) = (*x*·*y*)·*z* and *x*·1 = *x* = 1·*x*. This situation corresponds to an important algebraic structure, called a *monoid*. Restricting the category to just a single object introduces another example of category, where the self-directed arrows play the role of the elements, such as numbers, and composition plays the role of the binary operation, such as multiplication (Example 6).

**Example 6 (monoid)**. *A* *monoid* (*M*, ·, *e*) *consists of a set *M*, a binary operation ·, and an element* *e* ∈ *M satisfying*

- associativity: *a*·(*b*·*c*) = (*a*·*b*)·*c* and
- unity: *a*·*e* = *a* = *e*·*a*

for all elements *a, b, c* ∈ *M*. For instance, the set of real numbers together with elementary multiplication and 1 constitute a monoid, (ℝ, × , 1). The binary operation is called *multiplication*, and *e* is called the *unit*. These concepts are abstract notions. Any set and binary operation satisfying these axioms is a monoid, which includes the real numbers together with elementary addition as the “multiplication” operation and 0 as the “unit” element, (ℝ, +, 0). Every monoid corresponds to a one-object category whose elements *a* ∈ *M* correspond to the arrows *a*:* → * and binary operation to the composition operation, that is, *a*·*b* corresponds to the composite *b* ◦ *a*:* → *. The unit, *e*, corresponds to the identity arrow.

#### 2.1.6. Categories of structures as objects and structural relations as arrows

The examples introduced so far involved categories of objects that were elements or sets of elements with no other “internal” structure, that is, there were no relationships between the constituents of the objects themselves. However, an entity may have additional internal structure that requires modeling. For instance, similarity judgments may be regarded as a map on a set of related perceptual states so that similar stimuli evoke similar responses. One approach to this situation is to model the internal structure of an entity in the same way as domains are modeled, that is, as categories that are now objects in a “larger” category. Yet, this approach can be too restrictive as a collection of objects that are not categories individually may still constitute a category collectively. An alternative approach is to consider a weaker notion of structure, where the relations between constituents need not be reflexive (identity arrows are not required) or transitive (composition of arrows is not required). For instance, *parent-of* is neither reflexive, nor transitive. An efficient way of constructing categories for such situations is to use the internal structure of an existing category, which can circumvent the need to prove that the new construction satisfies the requirements of a category. A prototypical example is to construct the category of graphs and graph homomorphisms from the category of sets and functions, **Set** (Section 2.1.6). Constructing categories from a progenitor category, such as **Set**, can also be employed to model structural relations between domains of interest: If the constructed object is also a category, then maps between such objects correspond to maps between categories. A prototypical example of this situation is the category of monoids and monoids homomorphisms (Section 2.1.6). When the target object is a general category, this approach leads to a definition of map between category, called *functor*, which is taken up later (Section 2.1.6).

##### 2.1.6.1. Internal structure

A weaker notion of structure is *directed graph*, simply called *graph* hereafter, which consists of a set of *vertices*, a set of directed *edges* between vertices, and two maps determining the *source* and *target* vertex of each edge (Definition 7). Since a graph is given by sets and functions, graphs can be constructed from the internal structure of **Set**. A graph need not have a *loop* for each vertex (i.e., an edge whose source and target are the same vertex), or an edge for each (connected) *path*. A relation *R* on a set *A* corresponds to a graph with a vertex for each element *a* ∈ *A* and an edge from *a* to *a*′ for each relationship *aRa*′. Hence, graphs correspond to relations that need not be reflexive or transitive.

** Definition 7 (graph)**. *A* *(directed) graph* *G* = (*G*_0_, *G*_1_, *src, tgt*) consists of

- *a set of* *vertices*, *G*_0_ = {*v, w*, … },
- *a set of* *edges*, *G*_1_ = {*e, f*, … }—*an edge* *e* *is directed from a vertex* *v* *to a vertex* *w*, *written* *e*:*v*→*w*, *called the* *source* *and* *target* *of* *e*, *respectively, and*
- *two maps*, *src, tgt*:*G*_1_→*G*_0_ *sending each edge* *e*:*v*→*w* *to its source and target vertex, respectively, that is*, *src*(*e*) = *v* and *tgt*(*e*) = *w*.

A comparison of the definitions for graph and category makes clear that graph is indeed a weaker notion of structure than category—every category corresponds to a graph by regarding the objects as vertices and the arrows as edges; however, not every graph can be interpreted as a category, because a graph may not have a loop for each vertex corresponding to an identity arrow for each object, or an edge for each path corresponding to an arrow for each composition of arrows.

Perhaps less clear is whether a collection of graphs (as objects) constitutes a category. To clarify this point, we need to specify the relations between graphs (as arrows), how those relations compose (as the composition operation), and whether composition satisfies associativity and unity. (The other relations are usually easy to provide, that is, *dom*, *cod* and *id* maps.) The archetypal relation between graphs is *graph homomorphism*, that is, a pair of maps $\left(h_{0},h_{1}\right):G\to G^{\prime }$ sending each vertex *v* in *G* to the vertex *h*_0_(*v*) in *G*′ and each edge *e*:*v*→*w* in *G* to the edge *h*_1_(*e*):*h*_0_(*v*) → *h*_0_(*w*) in *G*′ (see Remark 8). The identity arrows are pairs of identity maps on the sets of vertices and edges. To show that this arrangement constitutes a category is straightforward, albeit tedious, as one needs to show composition produces a graph homomorphism satisfying associativity and unity.

A more efficient approach is to construct the graph from another category, which carries over the needed properties of associativity and unity, that is, to use the internal structure of another category. Observe that a graph is given by a pair of sets and a pair of functions. Thus, graphs are constructed from the internal structure of the category of sets and functions, **Set** (Remark 8).

**Figure 5:**
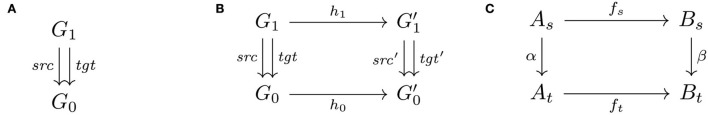
**(A)** Graphs, **(B)** graph homomorphisms, and **(C)** arrow homomorphisms are expressed as arrows.

** Remark 8**. A graph *G* = (*G*_0_, *G*_1_, *src, tgt*) can be expressed as a pair of arrows (Figure 5A). Hence, a graph homomorphism *h*:*G*→*G*′ is expressed by the *pair* of commutative squares (Figure 5B), that is, a pair of maps (*h*_0_, *h*_1_) such that $h_{0}◦src={src}^{\prime }◦h_{1}$ and $h_{0}◦tgt={tgt}^{\prime }◦h_{1}$.

This approach can be generalized further by making use of the analogy between vertices/edges and objects/arrows, that is, an arrow corresponds to a graph with domain and codomain objects corresponding to source and target vertices. We just saw how a graph is an object and an arrow between graphs (as objects) is a graph homomorphism. Accordingly, an arrow is now an object and an arrow between such objects is now an arrow homormorphism given by a commutative square of arrows in some category (example 9).

**Example 9 (arrows)**. *The category* ***Arr*(*C*)** *has for objects the arrows* α:*A*_*s*_→*A*_*t*_ of **C** *and for arrows the arrow homomorphisms* *f*:α → β, *that is, pairs of arrows* (*f*_*s*_, *f*_*t*_) of **C** *such that the corresponding diagram commutes (Figure 5C). The identities* 1_α_ in ***Arr*(*C*)** *are the pairs of identity arrows* (1_*A*_*s*__, 1_*A*_*t*__). *Composition in* ***Arr(C)*** *is composition of commutative squares, which follows from the associativity and unity properties of the composition operation*, ◦ , *in the original category* **C**.

This approach also applies to ordered sets. Recall (Section 2.1.3) that a strictly ordered set is not a category because it lacks reflexivity, hence the corresponding identity arrows. However, a collection of strictly ordered sets is a category in the same way as for graphs. A strictly ordered set is a special case of a graph that lacks loops or cycles, that is, edges or paths from/to the same vertex *v* corresponding to the relationship *v* ≤ *v*, and at most one edge from each source to each target. The objects are strictly ordered sets and the arrows are *monotonic functions*, that is, a function *f*:*P*→*Q* such that *p*<*p*′ implies *f*(*p*) < *f*(*p*′) for all elements *p* ∈ *P*. For comparison, a monotonic function on a non-strict ordered set, (*P*, ≤), satifies *p* ≤ *p*′ implies *f*(*p*) ≤ *f*(*p*′) for all *p* ∈ *P*.

##### 2.1.6.2. External structure

If the object of interest is already a category, then the maps between these objects constitute a notion of “external” category structure, that is, maps between categories, taken up in the next section (Section 2.2). For example, a monoid can also be expressed in terms of sets and functions (Example 10).

*Example 10 (monoids). Suppose monoids (*M*, ·_*M*_, *e*_*M*_) and (*N*, ·_*N*_, *e*_*N*_). A *monoid homomorphism* is a function *h*:*M*→*N* that preserves the*

- *binary operation: *h*(*a*·_*M*_*b*) = *h*(*a*)·_*N*_*h*(*b*) and*
- *unit: *h*(*e*) = *e*_*N*_*.

**Figure 6:**
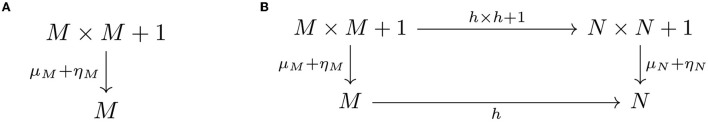
**(A)** Monoid and **(B)** monoid homomorphisms are expressed as arrows.

A monoid (*M*, ·, *e*) is equivalently given as the triple (*M*, μ, η), where μ:*M*×*M*→*M* is the binary operation expressed as a bivariate function, that is, μ:(*a, b*)↦*a*·*b*, and η:1 → *M* is the unit expressed as a nullary function, that is, η:*↦*e*. Thus, a monoid corresponds to a sum of arrows μ+η:*M*×*M*+1 → *M*, where the addition symbol signifies *disjoint union* of sets and functions, that is, *A*+*B* = {(1, *a*)|*a* ∈ *A*}∪{(2, *b*)|*b* ∈ *B*} with the corresponding sums of functions (Figure 6A), and a monoid homomorphism *h*:*M*→*N* corresponds to a commutative square (Figure 6B). The associativity and unity conditions for a monoid are also expressed as commutative diagrams.

### 2.2. Functors and representation

Like compositionality, some notion of *representation* is central to category theory and cognitive science. A cognitive representation is usually taken to mean a mental state that stands in some correspondence relation to a state of the world, which can include other mental states, and a compositional (cognitive) representation means that the relationships between constituent mental states correspond to relationships between the constituent states of the world. Suppose, for example, a state of the world that has *John is to the left of Mary*. Viewing cognition as a *language of thought* (Fodor, 1975), for instance, supposes at least a symbol for *John*, John, and a symbol for *Mary*, Mary, that are juxtaposed in such a way that the spatial relationship between *John* and *Mary* is expressed by the syntactic relationship between their corresponding symbols: e.g., the pair of symbols (John,Mary). These symbolic representations are supposed to map to corresponding brain states by a *physical instantiation mapping* that likewise preserves the corresponding relations (see, e.g., Fodor and Pylyshyn, 1988, footnote 9). Category was introduced as a form of compositionality. A map between categories is called a *functor* preserving categorical structure. Functors afford a category theory notion of compositional cognitive representation and instantiation.

The value of casting the definition of category as collections of objects and arrows and their structural relations in terms of maps between those collections now becomes apparent. A map between categories, that is, a functor, is straightforwardly just a homomorphism preserving those structural relations, specified by equality conditions (Definition 11).

** Definition 11 (functor)**. *Suppose categories **C** and **D**. A *functor* *F*:**C**→**D** is a pair of maps (*F*_0_, *F*_1_):(**C**_0_, **C**_1_) → (**D**_0_, **D**_1_) that preserves*

- *domains and codomains: *dom*(*F*_1_(*f*)) = *F*_0_(*dom*(*f*)) and *cod*(*F*_1_(*f*)) = *F*_0_(*cod*(*f*))*,
- *identities: *F*_1_(1_*A*_) = 1_*F*_1_(*A*)_ and*
- *compositions: *F*_1_(*g* ◦ *f*) = *F*_1_(*g*) ◦ *F*_1_(*f*)*

*for all objects *A* ∈ **C**_0_, arrows *f* ∈ **C**_1_ and pairs of compatible arrows (*f, g*) ∈ **C**_1_×**C**_1_*.

The equivalent commutative diagrams for the equality conditions (in definition 11) make plain that a functor is simply a (homo)morphism of the maps that constitute the structure of a category (remark 12).

**Figure 7:**
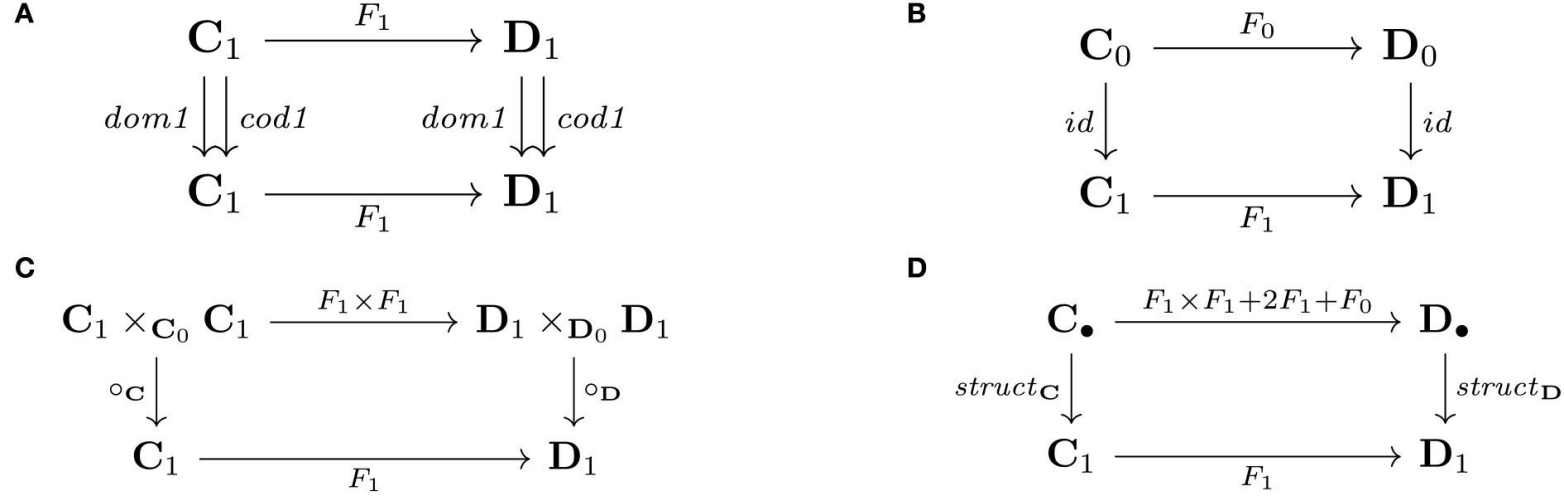
Functors as commutative squares preserving **(A)** domains/codomains, **(B)** identities, and **(C)** compositions, that is, **(D)** category structure.

** Remark 12**. *The conditions for a functor are equivalently given by commutative diagrams. The conditions for domains and codomains are given by commutative squares, where *dom1* = *id* ◦ *dom* and *cod1* = *id* ◦ *cod* (Figure 7A). The conditions for identity (Figure 7B), and composition (Figure 7C) are likewise expressed this way. So, a category is given as the sum of four maps that specify: (1) the composition operation, *comp*:**C**_1_×_**C**_0__**C**_1_→**C**_1_, (2) the domain of each arrow in the category as the associated identity arrow, *dom1*:**C**_1_→**C**_1_, (3) the codomain of each arrow in the category as the associated identity arrow, *cod1*:**C**_1_→**C**_1_, and (4) the identity arrow associated with each object in the category, *id*:**C**_0_→**C**_1_. The sum of these four arrows is the arrow *struct* = *comp*+*dom1*+*cod1*+*id*, where the addition symbol expresses alternative maps (see Example 14), that is, *struct*:**C**_•_→**C**_1_, where **C**_•_ denotes **C**_1_×_**C**_0__**C**_1_+**C**_1_+**C**_1_+**C**_0_. (**C**_1_+**C**_1_ is also written 2**C**_1_.) Categories **C** and **D** are given by arrows *struct*_**C**_ and *struct*_**D**_, respectively, and a map from *struct*_**C**_ to *struct*_**D**_ is a commutative square (Figure 7D). Hence, a functor is a category homomorphism*.

Since functors are expressed as commutative squares, they also compose in a way that satisfies associativity and unity conditions, that is, categories, functors, and functor composition constitute another category (Remark 13).

** Remark 13**. *Commutative squares compose as commutative squares, hence composition of functors *F*:**C**→**D** and *G*:**D**→**E** is a functor, *G* ◦ *F*:**C**→**E**. So, (small) categories, functors, and functor composition constitute another category, **Cat**. (In this context, *small* means that the collections of objects and arrows are sets, not proper classes.)*

A simple way of composing representations is to take products. Products are constructed from product functors (Example 14). For instance, assuming a set of symbols for *John* and *Mary*, *S* = {John,Mary}, the product functor constructs the set of symbol pairs *S*×*S* = {(John,John), (John,Mary), … }, which can be used for to represent the *John is to the left of Mary* situation.

**Example 14 (product/coproduct functors)**. *The *product* and *coproduct functors* send pairs of objects and arrows to their products and coproducts, respectively, that is, Π:(*A, B*)↦*A*×*B*, (*f, g*)↦*f*×*g* and ∐:(*A, B*)↦*A*+*B*, (*f, g*)↦*f*+*g*. In the category of sets and functions, **Set**, the product of two sets is (designated as) their Cartesian product and the product of two functions *f*:*A*→*C* and *g*:*B*→*D* is the *product function*, *f*×*g*:(*a, b*)↦(*f*(*a*), *g*(*b*)). The coproduct of two sets is (designated as) their disjoint union, and the coproduct of two functions *f* and *g* is the *coproduct function*, *f*+*g*:*A*+*B*→*C*; (1, *a*)↦*f*(*a*), (2, *b*)↦*g*(*b*). The coproduct for sets and functions acts like alternation: if *a* is an element from set *A*, then apply function *f*:*A*→*C*, otherwise apply function* *g*:*B*→*C*.

A product functor takes objects in a category **C** to (product) objects in **C**. In this way, constructions can be reiterated to generate compositional representations whose constituents are themselves compositional as is supposed for a language of thought. For instance, *Sue is to the left of John who is to the left of Mary* may be represented by a construction that involves the pair (Sue, (John,Mary)) capturing a hierarchical relationship, that is, *Sue* is to the left of both *John* and *Mary*. A general approach to this situation involves *monoidal categories* (Mac Lane, 1998; Leinster, 2014), that is, a generalization of monoid where the set is replaced with a category and the binary operation with a functor. In this context, the functor is a kind of *tensor product* (Example 15), which affords a categorical (symbolic-vectorial) form of compositional representations (Coecke et al., 2010). Connectionist (neural network) models employing *tensor product networks* (Smolensky, 1990) essentially involve such functors for a category of vectors spaces and linear functions.

**Example 15 (tensor product)**. *A *tensor product* is a functor of the form *F*:**C**×**C**→**C**, also written ⊗(−, −) with the action on a pair of objects (*A, B*) and a pair of arrows (*f, g*) written *A*⊗*B* and *f*⊗*g*, respectively. Associativity and unity conditions need only hold up to *natural isomorphism* (see Definition 16), that is, *A*⊗(*B*⊗*C*)≅(*A*⊗*B*)⊗*C* and *A*⊗*I*≅*A*≅*I*⊗*A*, where *I* is a special object in **C** whose role is analogous to the role of the unit element, *e*, of a monoid. For instance, product and coproduct functors (Example 14) are tensor products: in regard to **Set**, now seen as a monoidal category, the Cartesian product and disjoint union with any one-element set and the empty set as the unit objects, respectively*.

Entities and their representations are also naturally regarded as residing in different domains and so involve functors between different categories. For instance, the aforementioned notion of a physical instantiation mapping of symbolic expressions to brain states is likened to a functor that preserves syntactic relations as relations between brain states, that is, a mapping *F* that satisfies the equality *F*[*P&Q*] = *B*(*F*[*P*], *F*[*Q*]), where *B* is a function combining the corresponding brain states for expressions *P* and *Q* (see Fodor and Pylyshyn, 1988, footnote 9). This condition compares with the composition condition for functors, *F*_1_(*g* ◦ _**C**_*f*) = *F*_1_(*g*) ◦ _**D**_*F*_1_(*f*), where & corresponds to composition operation ◦ _**C**_ in domain category **C** and *B* corresponds to composition operation ◦ _**D**_ in codomain category **D**. The expressions *P* and *Q* and their corresponding brain states, *F*[*P*] and *F*[*Q*], are arrows in their respective categories. Alternatively, the expressions and brain states can be regarded as objects in a monoidal category, whence the mapping is seen as a *monoidal functor* (Mac Lane, 1998), that is, a functor preserving the structure of a monoidal category.

Much more can be said about a functorial approach to cognitive representation by specializing to functors with additional properties. For instance, an *adjoint functor* (Mac Lane, 1998; Leinster, 2014) is a functor *F*:**C**→**D** that comes with an opposing functor *G*:**D**→**C** acting as a pseudo-inverse in the sense that the composition *G* ◦ *F* sends objects and arrows in **C** to objects and arrows in **C** that are closely related but not necessarily the same as the original objects and arrows. (Likewise, the composition *F* ◦ *G* sends objects and arrows in **D** to closely related objects and arrows in **D**.) That relationship is a *natural transformation*, which we turn to next (Section 2.3). This form of bidirectionality has applications, for example, in regard to the round-trip relationship between states of the world and brains states (see Ellerman, 2016; Awodey and Heller, 2020). Another example of this adjoint situation in the context of cognitive representations and processes involves *presheaves* (Mac Lane and Moerdijk, 1992) that are set-valued functors on topological spaces as categories, where the round-trip relationship acts like a generalization process in the sense that learning a training set extends (generalizes) to correct responses on a test set (Phillips, 2018, 2020). Adjoint situations involve additional category theory concepts, that is, *universal constructions* (Mac Lane, 1998; Leinster, 2014), that go beyond the detailed comparisons presented here (see Section 3).

### 2.3. Natural transformations and comparison

Cognitive processes are generally regarded as computational processes over (cognitive) representations. Natural transformations are maps between functors, functors were interpreted as representations, so natural transformations can be interpreted as computational processes on representations. As we shall see in this section, however, natural transformations also afford a closely related interpretation as comparisons of representations.

** Definition 16 (natural transformation, isomorphism)**. Suppose *F, G*:**C**→**D** are a pair of functors. A *natural transformation* $\eta :F\overset{.}{\to }G$ is a family of **D**-arrows {η_*A*_:*F*(*A*) → *G*(*A*)|*A* ∈ *C*_0_} such that *G*(*f*) ◦ η_*A*_ = η_*B*_ ◦ *F*(*f*) for all arrows *f*:*A*→*B* in **C**, which can be expressed as a commutative square (Figure 2). Arrow η_*A*_ is called the *component* of η at *A*. If every component η_*A*_ is an *isomorphism*—arrow *f*:*A*→*B* is an isomorphism if there exists an arrow *g*:*B*→*A* such that *f* ◦ *g* = 1_*B*_ and *g* ◦ *f* = 1_*A*_—then the transformation is called a *natural isomorphism*.

The definition says that a natural transformation is composed of a family of maps from the image of one functor to the image of another functor. Hence, a natural transformation can be interpreted as a computational process for transforming representations. However, the family of maps is also required to satisfy the commutativity condition involving a square of arrows. This condition means that the transformed representations must also be comparable to the original representations. Two simple examples illustrate this situation (Example 17).

**Figure 8:**
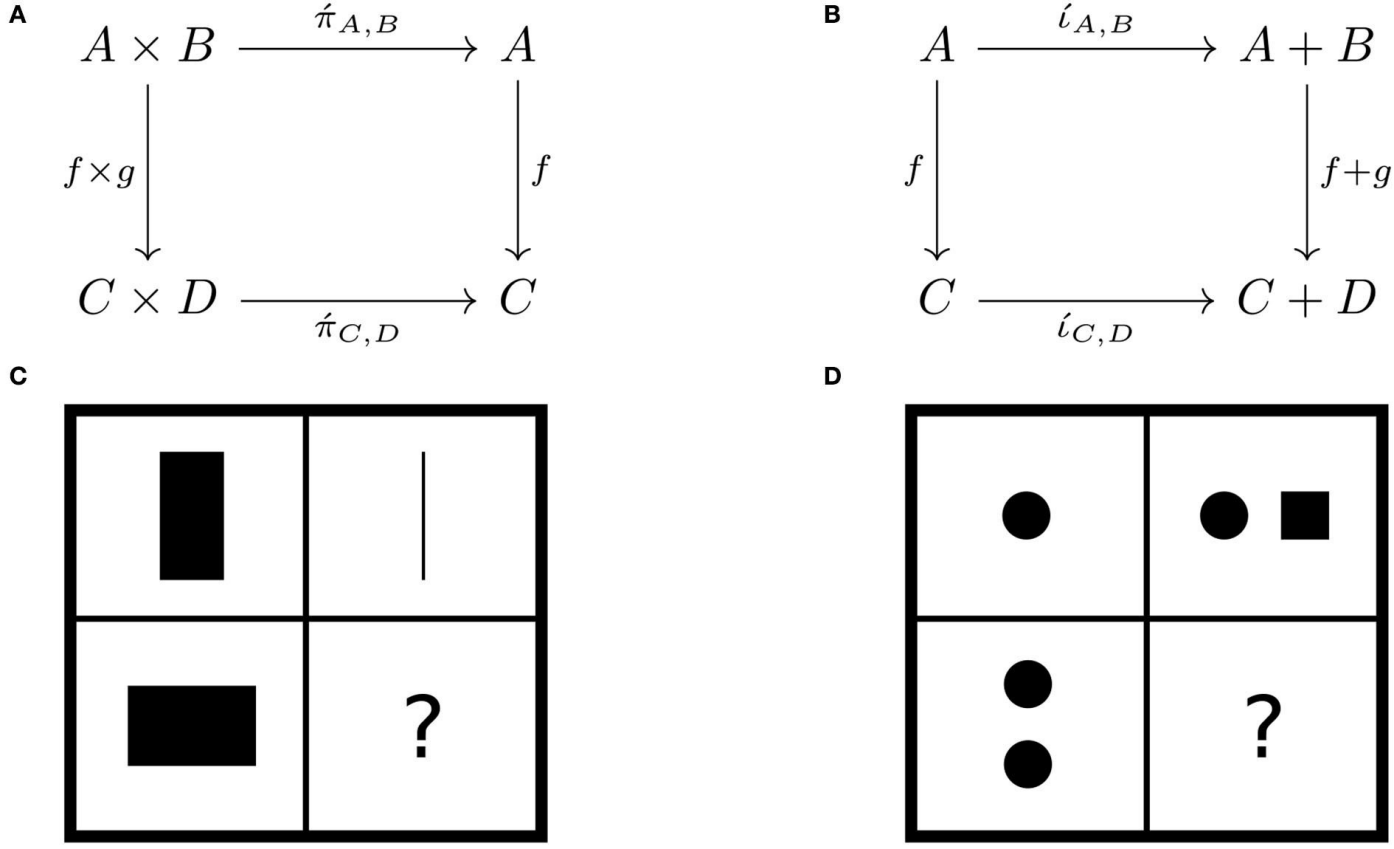
Two natural transformations: **(A)** natural projection and **(B)** natural injection as progressive matrices **(C,D)**, respectively.

**Example 17 (natural projections/injections)**. *The* *projection functors* *send pairs of objects and arrows to their components: e.g.*, $\overset{´}{\Pi }:\left(A,B\right)\mapsto A,\left(f,g\right)\mapsto f$. *Product, coproduct, and projection functors are related by natural transformations: e.g.*,

- *natural projection* $\overset{´}{\pi }:\Pi \overset{.}{\to }\overset{´}{\Pi }$ (Figure 8A) and
- *natural injection* $\overset{´}{\iota }:\overset{´}{\Pi }\overset{.}{\to }∐$ (Figure 8B).

*Projections and injections are conceptualized by analogy to instances of matrix reasoning (Figures 8C,D), which highlights the interpretation of natural transformation as a comparison of representations*.

**Figure 9:**
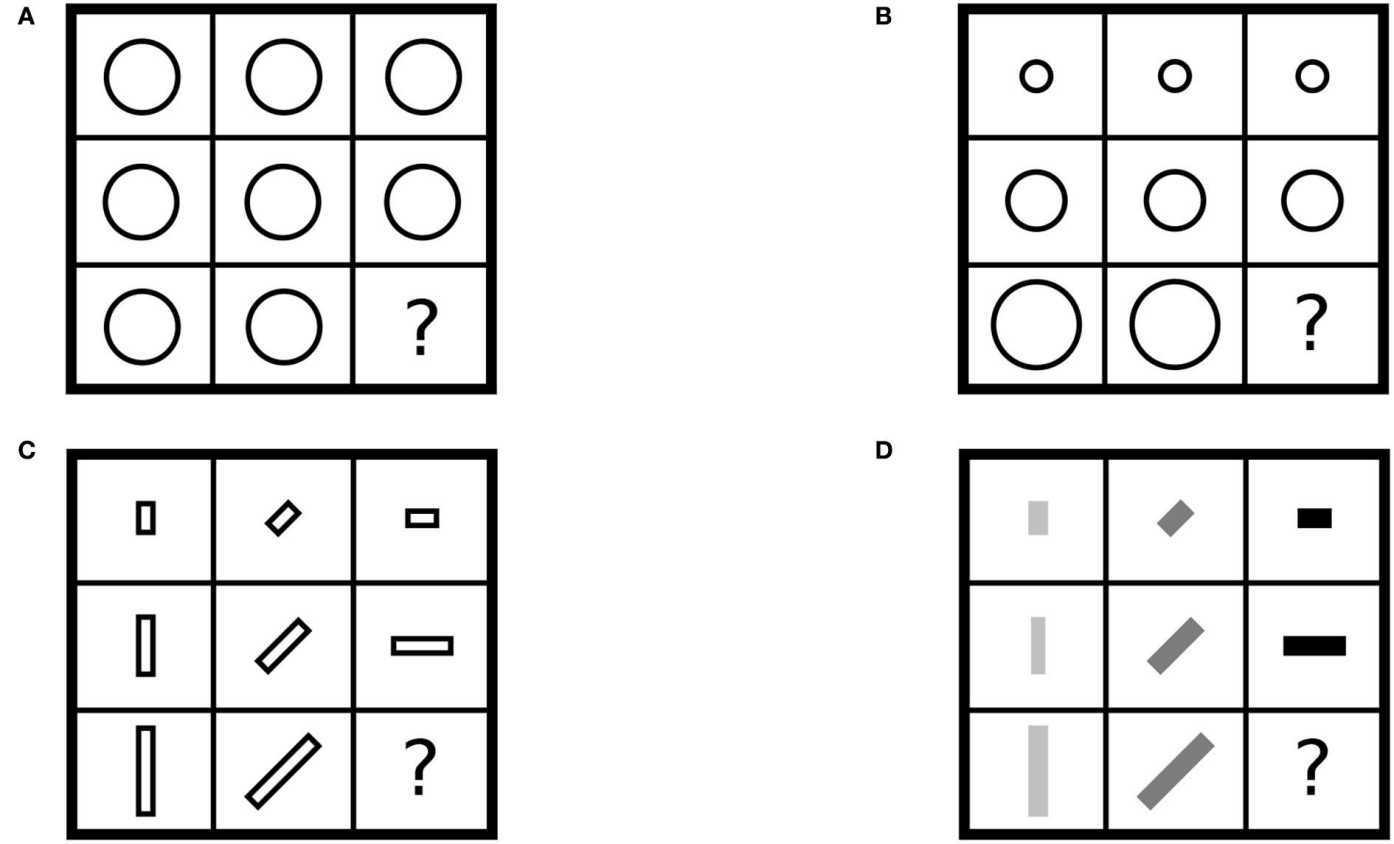
Dimensions of variation in matrix reasoning: **(A)** zero, **(B)** one, **(C)** two, and **(D)** three.

Recall (Section 1) that composition operates at all levels: composition of arrows (between objects), functors, and natural transformations. These levels also pertain to another notion of dimensionality that is illustrated with a series of progressive matrices (Figure 9). Identity arrows are interpreted as zero-dimensional in terms of variation, that is, the domain and codomain objects are the same object. Hence, there is a corresponding sense of dimensionality with progressive matrices in terms of stimulus variation along rows and columns of the matrix. For instance, when all cells contain the same stimulus (Figure 9A), the number of dimensions of variation is zero. This situation corresponds to an identity natural transformation on a *constant functor*, that is, a functor sending every object and every arrow to the same object and its identity arrow. A single dimension of stimulus variation along columns (Figure 9B) corresponds to an identity natural transformation on a functor that is not a constant functor. Alternatively, a single dimension of stimulus variation along rows corresponds to a non-identity natural transformation on a constant functor. Two dimensions of stimulus variation, along rows and columns (Figure 9C), correspond to a (non-identity) natural transformation of (non-constant) functors. Three dimensions of stimulus variation, one dimension along columns and two dimensions along rows (Figure 9D), correspond to composition of a functor with a natural transformation, which is a natural transformation. Composition of a functor with a natural transformation is also called *star composition* to distinguish this form from composition of natural transformations and composition of functors.

**Figure 10:**
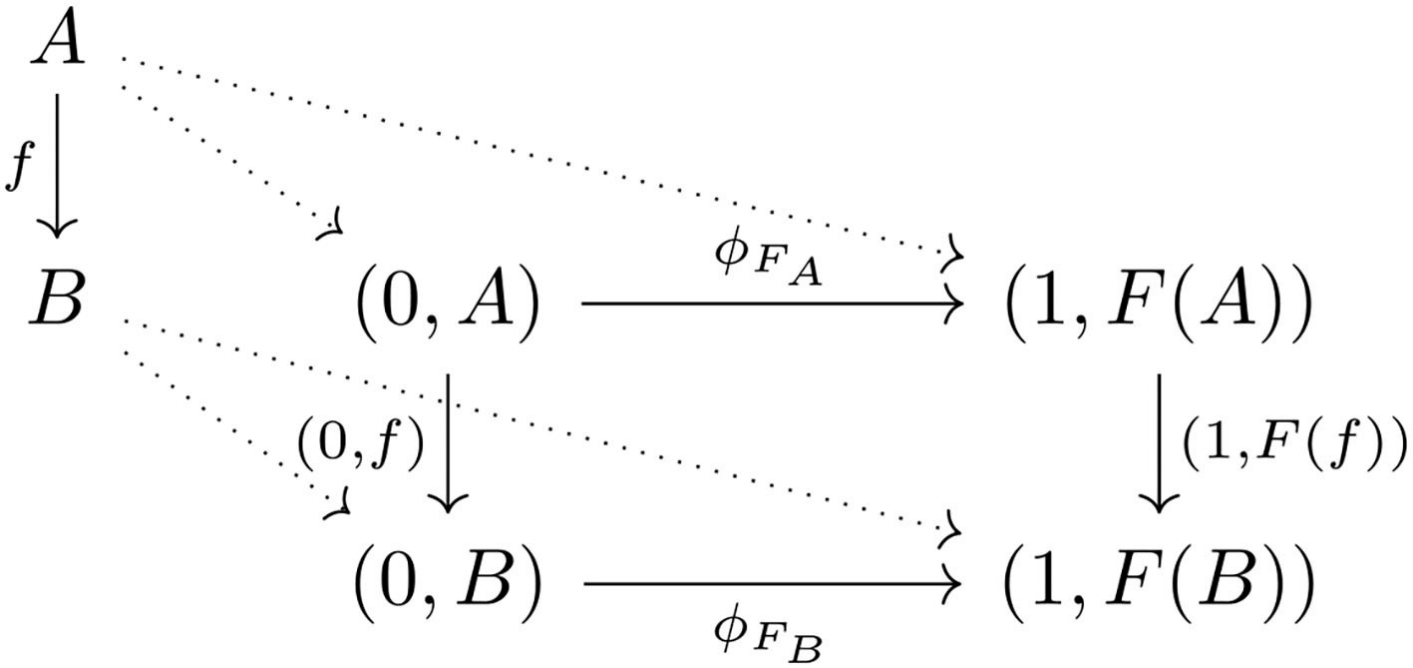
Functor as natural transformation.

Natural transformations mark a significant departure from functors in terms of dimensionality. A functor also involves a square of arrows (see Figure 2A), hence looks like a natural transformation. Indeed, every functor *F*:**C**→**D** is equivalent to a natural transformation between functors into **C**+**D** comparing the domain and codomain of *F* (Figure 10), that is, the natural transformation $\phi_{F}:\iota_{0}\overset{.}{\to }\iota_{1}•F$, where ι_0_:*A*↦(0, *A*), *f*↦(0, *f*) and ι_1_ ◦ *F*:*A*↦(1, *F*(*A*)), *f*↦(1, *F*(*f*)). However, the injection of the domain is effectively an identity map, hence involves no variation of objects and arrows along this direction. Thus, in general, natural transformations involve an extra dimension of variation compared to functors.

The examples of progressive matrices involve geometrical shapes, which highlight another aspect of category theory—generality. Up to this point, the examples have been about orders or simple algebraic structures. However, categories also exist for other kinds of structures including topological, metrical, and geometrical spaces. For instance, the category **Vec** has vector spaces for objects and linear maps for arrows. Thus, we can think of the shapes in terms of vector spaces and the transformations as linear maps such as dilation and rotation. The correspondence to categories, functors, and natural transformations is analogous, that is, the columns are functors from some category into **Vec** with shapes corresponding to vectors and arrows to linear transformations; likewise for the components of a natural transformation.

## 3. Discussion

What is category theory to cognitive science? In a broad sense, category theory like cognitive science is about the (re-)representation and comparison of compositional structure *via* maps that preserve that structure. Category theory may appear as a bewildering array of abstract definitions, examples, and theorems, but a substantial amount of this theory organizes around a simple idea, that is, the (typed) commutative square, in various contexts and forms, that comports with the view of cognition as a system of computational processes over (cognitive) representations, that is, some version of a representational/computational theory of mind (Wilson, 1999).

Representational (mental) states are supposed to capture the structure of the world, or other mental states by structural correspondence, that is, the relations between entities in the domain being represented are supposed to map in some consistent way to relations between entities in the domain of representations (cf. Frege's *compositionality principle*, or Gentner's *structure mapping theory*). A commutative square embodies this idea in geometric-algebraic form, that is, a “vertical” arrow is a structural relation in one domain that is transported, or transformed to a vertical arrow as a structural relation in another (possibly identical) domain by “horizontal” arrows that maintain structural consistency—the action on some (re-)representation is essentially the same as a (re-)representation of an action. The conditions for being a category, functor, or natural transformation mean that not any square of typed entities constitutes a commutative square. Category theory provides a vast formal generalization of this simple idea in a way that is unique among formal frameworks.

Category theory differs from other frameworks in regard to the notion of compositionality. The classical form of compositionality turns on the notion of *tokening*, that is, the representations of an entity's constituents are tokened (instantiated, activated, or inscribed) whenever the entity's representation is tokened (Fodor and Pylyshyn, 1988). For instance, a classical representation of *red circle* involves the tokening of corresponding representations for constituents *red* and *circle*. A composition operation need not token arrows in this way, as illustrated in the case of monoids where the composition of arrows 1 and 2 is their addition 3, because composition is a function that sends a pair of arrows to an arrow. A category theory analog of tokening is the *free monoid* on a set of characters (alphabet) A which consists of the set of all strings of zero or more characters, $A^{*}$, that is, the monoid $\left(A^{*},\cdot ,\epsilon \right)$, where · is concatenation and ϵ is the empty (length zero) string: e.g., composition of strings (arrows) “a” and “b” is the string “ba” (Walters, 1991).

Other (related) notions of compositionality were alluded to in the form of products and coproducts of objects and arrows: e.g., the product functor applied to two objects *A* and *B* constructs the product object *A*×*B*. What that means precisely depends on the categories involved and the specific choice of object. For instance, in **Set**, a product of sets and *A* and *B* is designated as their Cartesian product, also written *A*×*B*, and two functions affording the retrieval of the constituents of each pair (*a, b*), that is, *a* and *b*. There may be more than one product for a pair of objects, for example, *B*×*A* also constitutes a product for *A* and *B*. These examples seem to suggest that this form of categorical compositionality is a version of classical compositionality in that the constituent objects *A* and *B* are “tokened” with the composite object *A*×*B*. However, other products of sets exist that do not involve constructing pairs of constituent elements. This difference is starker in the context of ordered sets as categories where the product of two objects *A* and *B* is the *infimum*, that is, the greatest object less than both *A* and *B*. For example, in an ordered set as a category of *divisors* (e.g., 2 → 6 says *two is a divisor of six*), the categorical product of 12 and 9 is the greatest common divisor, that is, 3. Category theory provides a precisely defined and vastly generalized notion of this form of composition that involves the concepts of *limit* and *universal construction* (Mac Lane, 1998; Leinster, 2014). A limit is a kind of “optimal” construction, that is, the best one can do in the given context and also a universal construction expressing a property common to all instances in that context (see Phillips, 2021a, for an introduction in the context of cognition). Though not taken up here, *systematicity* (Fodor and Pylyshyn, 1988), that is, the co-existence of cognitive abilities, is seen as a consequence of a (categorical) universal construction (Phillips and Wilson, 2010).

The import of category theory concepts to cognitive science does not end there. Base concepts of category, functor, and natural transformation constitute the starting point for category theory like the concepts of composition, representation and computation (comparison) constitute a starting point for a science of cognition. Natural transformations afford inference as do comparisons of representations. An analogy to perception provides an illustration. Inferring distance to an object is afforded by comparison of images obtained from binocular vision. A category theory analog is the *Tannakian reconstruction theorem* affording reconstruction of an object from its category of representations (NLAB, 2010). Underpinning this theorem is the *Yoneda lemma* (Mac Lane, 1998; Leinster, 2014), a fundamental result in category theory (Riehl, 2016) that relates the structure of an object to its afferent/efferent arrows. The reconstruction theorem was applied to the *relational schema induction* paradigm (Halford et al., 1998a) to account for learning transfer (Phillips, 2021b). Reconstruction involves a higher dimensional form of comparison, called a *dinatural transformation* between *bifunctors*, which are functors on two categories (cf. bivariate function). Inference is afforded by a *duality*—two opposing relations—between schemas (more generally algebras) and their representations that is analogous to a well-known duality in geometry: Two points determine a line; dually, two (intersecting) lines determine a point. For comparison, source and detection determine line of sight; dually, intersecting lines of sight determine the source. Reconstruction involves computing the *end* of a (bi)functor (Mac Lane, 1998), that is, the “best” (universal) higher dimensional comparison.

Higher dimensional constructions relate to higher cognitive capacities, as alluded to in the comparison of matrix reasoning examples. Matrix reasoning is generally more difficult when the stimuli vary along more dimensions (Carpenter et al., 1990; Kroger et al., 2002) corresponding to a notion of cognitive complexity as the number of dimensions of task variation (Halford et al., 1998b), which has been interpreted in terms of categorical products (Phillips et al., 2009). Note that category, functor and natural transformation also have corresponding geometrical interpretations as points, lines, and sheets, hence as zero-dimensional, one-dimensional, and two-dimensional objects, respectively. Indeed, these concepts unify in higher category theory as instances of *n cells*: e.g., a *2-category* that has (small) categories as 0 cells (objects), functors as 1 cells (arrows between 0 cells), and natural transformations as 2 cells (arrows between 1 cells). This notion of dimensionality is akin to the *order* of a function or relation—a second-order relation is a relation between (first-order) relations—as another measure of cognitive complexity (Zelazo and Frye, 1998).

The import of category theory to cognitive science may seem obscured by the many technical details that could be relegated to a secondary source. However, what counts as the primary focus of attention depends on the task at hand. Moving to higher constructions is not about simply affording more general generalizations (abstractions), but rather reconciling a seemingly opposed need for concreteness. This situation is exemplified with the associativity and unity conditions for composition, which seem innocuous when viewed as counterparts in elementary algebra, but are of fundamental importance to category theory and by comparison cognitive science. For instance, the associativity condition is recovered from a natural transformation between *hom-functors* (Mac Lane, 1998; Leinster, 2014), which determine the afferent/efferent arrows for a given object in the category of interest. The clockwise and anticlockwise traversals of the commutative square for the natural transformation between hom-functors correspond to the two alternative orders of composition, that is, *h* ◦ (*g* ◦ *f*) vs. (*h* ◦ *g*) ◦ *f*. A cognitive counterpart concerns dual-route theories (Kahneman, 2011; Evans and Stanovich, 2013), simply illustrated here by the relative difficulty of calculating (13 × 47) × 0 vs. 13 × (47 × 0), which affords a category theory way of thinking about and empirically investigating dual-route cognitive processes (Phillips et al., 2016, 2017). The identity arrows for the unity condition are also of fundamental importance despite appearances as the “do nothing” arrows. Tannakian reconstruction depends on the Yoneda lemma which in turn depends on having identity arrows. The identity arrows, appearing as elements in a hom-set of arrows for the proof of the lemma (see, e.g., Leinster, 2014), essentially ground the abstraction, cf. Frege's *principle of contextuality*, whereby the meaning of a word is determined in the context of other words (Janssen, 2001). For instance, the Yoneda lemma is seen as a way of assessing a subjective experience by its relationships to other subjective experiences (Tsuchiya and Saigo, 2021). This Yoneda/Tannaka perspective suggests an addendum to the import of category theory to cognitive science as self-referential comparison.

## Author contributions

The author confirms being the sole contributor of this work and has approved it for publication.

## Funding

This work was supported by a Japanese Society for the Promotion of Science Grant-in-Aid (20H05710).

## Conflict of interest

The author declares that the research was conducted in the absence of any commercial or financial relationships that could be construed as a potential conflict of interest.

## Publisher's note

All claims expressed in this article are solely those of the authors and do not necessarily represent those of their affiliated organizations, or those of the publisher, the editors and the reviewers. Any product that may be evaluated in this article, or claim that may be made by its manufacturer, is not guaranteed or endorsed by the publisher.
